# Actinomycosis

**Published:** 1886-10

**Authors:** 


### ACTINOMYCOSIS.

In another column appears the report of a case of this comparatively rare disease in the human subject. The number of cases thus far reported is about thirty-six; the great majority having occurred in Germans of the peasant class. It would seem as if their habits peculiarly exposed them to infection from this fungus. This last case, it will be noted, is a German, and, from the history of the case, seems to have contracted the disease in his native land. A very interesting point in the history is the length of time, seven years, that has elapsed since the primary infection, and the manner in which the fungus gained access to the tissues. Two cases reported during the past year bear directly on this point: the first, by I. Israel (*Central blat. für Med. Wissensch.*), is that of a coachman from Russia, who used the hay-loft of his barn as a bedchamber, and often drank from his horses' bucket. He first complained of a severe pain in the left side, and shortly thereafter an abscess appeared, the pus of which contained a large number of actinomyces. An examination of the lungs after death revealed a large mass of actinomyces in the anterior inferior portion of upper lobe of the left lung. In the centre of this mass a piece of a tooth was found. Israel advances the hypothesis that lung-actinomycosis is brought about by the aspiration of the infectious material from the mouth, and that sometimes this fungus is developed on the teeth.

The second case, by Professor Soltmann (*Jahrb. für Kinderhkde, 1886*), was a boy who had swallowed an ear of fox-grass ("*tauben Gerste*") which after a few days was discharged in the contents of a small abscess at the angle of the right scapula. Some six months after this accident the boy presented another swelling to the right of the vertebra which contained pus and actinomyces. Soltmann regards this as confirming Ponfick's view that plants may act as the vehicle of infection.

While the history of Dr. Schirmer's case is imperfect, yet in the light of the above facts, the slight wound of the chin may be regarded as the source of infection, which may have occured at the time of its reception or later.

